# Concomitant Panniculectomy in Abdominal Wall Reconstruction: A Narrative Review Focusing on Obese Patients

**DOI:** 10.3390/clinpract14020052

**Published:** 2024-04-22

**Authors:** Salvatore Giordano, Andre’ Salval, Carlo Maria Oranges

**Affiliations:** 1 Department of Plastic and General Surgery, Turku University Hospital, University of Turku, 20014 Turku, Finland; a.salval@istitutoesteticoitaliano.it; 2 Department of Plastic, Reconstructive and Aesthetic Surgery, Geneva University Hospitals, University of Geneva, 1205 Geneva, Switzerland; carlo.oranges@hcuge.ch

**Keywords:** obesity, panniculectomy, hernia, abdominal wall, surgical mesh, postoperative complications, BMI

## Abstract

The global prevalence of obesity continues to rise, contributing to an increased frequency of abdominal wall reconstruction procedures, particularly ventral hernia repairs, in individuals with elevated body mass indexes. Undertaking these operations in obese patients poses inherent challenges. This review focuses on the current literature in this area, with special attention to the impact of concomitant panniculectomy. Obese individuals undergoing abdominal wall reconstruction face elevated rates of wound healing complications and hernia recurrence. The inclusion of concurrent panniculectomy heightens the risk of surgical site occurrences but does not significantly influence hernia recurrence rates. While this combined approach can be executed in obese patients, caution is warranted, due to the higher risk of complications. Physicians should carefully balance and communicate the potential risks, especially regarding the increased likelihood of wound healing complications. Acknowledging these factors is crucial in shared decision making and ensuring optimal patient outcomes in the context of abdominal wall reconstruction and related procedures in the obese population.

## 1. Introduction

Obesity prevalence is continuously increasing worldwide, affecting 10 to 30% of adults [1]. It is defined as a condition where weight exceeds the ideal by over 20% or a body mass index (BMI) over 30 kg/m^2^. Considered a chronic, multifactorial disease, it involves genetic, environmental, psycho-social, biologic, neurologic, and cultural factors resulting in an excess of adipose tissue. Multiple diseases are associated with obesity, primarily including type II diabetes, gallbladder disease, hypertension, hyperlipidemia, coronary artery disease, cancers, osteoarthritis, depression, sleep apnea, incontinence, and infertility [2]. Consequently, obesity itself increases the risk of complications and mortality in various surgical interventions [3]. It has been calculated that obesity is the cause of death for 4 million people annually worldwide, representing a significant burden on healthcare systems and social services due to associated costs. In this context, abdominal wall reconstruction (AWR), which involves the repair of ventral hernias and/or oncological defects, presents significant challenges in obese patients, despite advances in surgical techniques and mesh materials. Notably, the incidence of abdominal or ventral hernias is higher in obese or overweight individuals. Obesity is an independent risk factor not only for the development of primary hernias but also for their recurrence [4]. Recent estimates suggest that over 350,000 ventral hernia repairs are performed annually in the United States, incurring costs exceeding 3 billion dollars [5].

Reducing complications in AWR for obese patients is challenging, as these individuals often have multiple comorbidities associated with obesity. The excision of excess skin and adipose tissue overlying the abdomen is an attractive option for surgeons. It enhances visibility during hernia or abdominal wall defect repair and potentially improves postoperative outcomes by reducing tension on the surgical site [6]. Patients may also be highly satisfied with the improved abdominal contour. However, procedures concurrent with AWR, such as panniculectomy, appear to result in higher surgical site morbidity [6,7].

Only a few studies have addressed the impact of panniculectomy on AWR, especially in obese patients. This narrative review aims to present the current outcomes of concomitant panniculectomy with AWR in obese patients. We hypothesized that concomitant panniculectomy might increase surgical site morbidity without affecting the rate of hernia recurrence. 

## 2. Materials and Methods

This narrative review is dedicated to elucidating and discussing the latest findings regarding abdominal wall reconstruction (AWR) in obese patients. Extensive literature exploration was conducted to unearth pertinent sources pertaining specifically to panniculectomy within the broader realm of AWR. Medline, Cochrane Library, Embase, Scopus, and Google Scholar databases were rigorously interrogated. The search was meticulously tailored to identify studies focusing on outcomes associated with AWR and panniculectomy. Utilizing Boolean search terms such as “abdominal wall reconstruction”, “hernia repair”, “panniculectomy”, “abdominoplasty”, and “morbid obesity”, the search strategy aimed to capture a comprehensive array of relevant studies for a comprehensive analysis and discussion.

Studies to be included in this review had to match predetermined criteria according to the PICOS (patients, intervention, comparator, outcomes, and study design) approach. Criteria for inclusion and exclusion are specified in Table 1. No limitations were applied on ethnicity, age of patients, or geographical area. Two authors (SG and CMO) assessed the abstracts and articles independently. Additionally, the reference lists of relevant articles underwent thorough examination. This analysis focused on eligible studies that reported outcomes following panniculectomy and AWR.

Each study underwent independent evaluation by two co-authors (SG and CMO) to determine inclusion or exclusion from the analysis (refer to Table 1). To be included, studies had to provide details on baseline characteristics, the type of procedure, the surgical technique used, and outcomes related to postoperative complications, particularly the hernia recurrence and surgical site occurrence (SSO). The research was limited to articles published in English from inception to December 2023, and only clinical studies were considered for inclusion.

A recurrent hernia was considered, as reported by the included studies, typically presenting as a contour abnormality associated with a fascial defect. Surgical site occurrence was defined as any complication involving the abdominal wall, encompassing infections, wound dehiscence, fat necrosis, hematomas, and seromas.

## 3. Results

All relevant articles identified were thoroughly examined for inclusion in this narrative review. The search yielded a randomized controlled trial [8], two prospective studies [9,10], and fourteen retrospective studies [11,12,13,14,15,16,17,18,19,20,21,22,23]. Among them were two propensity score analyses [6,23]. These studies varied considerably in their reported surgical techniques for abdominal wall reconstruction (AWR) and hernia repair. In most instances, a mesh—predominantly synthetic—was utilized, though the specific plane of placement was not consistently detailed. The mean body mass index (BMI) reported in these studies consistently exceeded 30 kg/m^2^, underscoring the prevalent incidence of obesity among the patient populations studied. Obesity classes are defined as class I obesity (BMI: 30.0–34.9 kg/m^2^); class II obesity (BMI: 35.0–39.9 kg/m^2^); class III obesity (BMI: 40.0–49.9 kg/m^2^); super-obese (BMI > 50.0 kg/m^2^).

### 3.1. Obesity and AWR

It is well recognized that obesity is linked to various surgical and medical complications, which intensify as the BMI increases across different obesity classes [4,7]. In the open technique, the most common complication is surgical site occurrence, escalating from 14.9% in class I obesity (BMI: 30.0–34.9 kg/m^2^) to 36.8% in class II/III obesity (BMI > 35.0 kg/m^2^) [4]. Interestingly, hernia recurrence rates do not appear to be significantly influenced by the obesity class, remaining between 7.7% and 10.3% from class I to II/III. This is echoed in findings from the American College of Surgeons National Surgical Quality Improvement Program (NSQIP), which showed an increase in surgical complications from 9.7% in obese to 19.9% in super-obese patients (BMI over 50 kg/m^2^), with the odds ratio for any surgical complication ranging from 1.22 to 2.66 in the super-obese category [22]. Supporting this, another study using the NSQIP database revealed that post-ventral hernia repair complications were more likely in patients with a BMI over 40 kg/m^2^, notably with a 28.7% rate of recurrent repair [24,25]. Outcomes in obese patients seem to be adversely impacted regardless of the surgical technique used for AWR. Laparoscopic ventral hernia repair may offer a lower risk of surgical site occurrences (SSOs) and a shorter hospital stay compared to the open approach [26,27]. However, this technique is not as widely adopted, possibly due to concerns about intraperitoneal mesh placement and intrafascial suturing not ensuring a functional restoration of the abdominal wall [26,27]. More recently, the adoption of the robotic approach for ventral hernia repair has been on the rise, showing promising results. However, long-term follow-up data on hernia recurrence rates are still needed [28,29,30,31]. Therefore, while minimally invasive techniques may reduce SSOs and hospital stays, their long-term efficacy in obese patients requires further validation.

### 3.2. Panniculectomy and AWR

**Figure 1:**
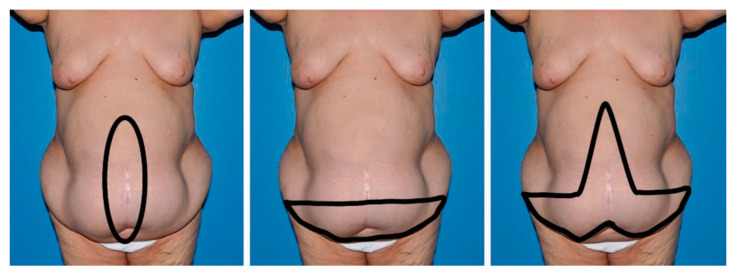
Schematic representations of the different types of panniculectomy. (**left**) Vertical, (**center**) horizontal, and (**right**) fleur-de-lis. This figure is taken from Giordano et al. [6].

Panniculectomy, defined as the removal of excessive skin and subcutaneous fat tissue from the abdominal wall, is indicated when the excess is over 4 cm in size [6]. This procedure can be executed in three distinct styles, vertical, horizontal, or fleur-de-lis, selected based on the patient’s specific needs and characteristics (Figure 1). Abdominoplasty was not performed in the included studies.

This surgery is particularly beneficial when there is a significant excess of skin and subcutaneous fat on the abdominal wall. It not only enhances surgical exposure during abdominal wall reconstruction (AWR) but also alleviates the tension on the skin post-surgery. Additionally, panniculectomy facilitates the removal of excess tissue following extensive dissections due to hernia manipulations and/or component separation techniques. Beyond its functional benefits, the procedure often leads to aesthetic enhancement in the abdominal contour, thereby increasing patient satisfaction.

A pooled analysis investigated the complications in patients undergoing panniculectomy in conjunction with AWR [7]. The reported pooled hernia recurrence was 4.9%. The most frequent complication identified was surgical site occurrence, with incidences ranging from 15 to 47 percent (pooled: 27.9%). The most prevalent specific complication was wound infection (16%). Other complications reported include seromas (11%), surgical wound dehiscence (11%), delayed wound healing (6%), skin necrosis (4.5%), and hematomas (0.4%). The overall incidence of medical and systemic complications was estimated at 8%, predominantly involving a thromboembolism (1.2%). Recently, Diaconu et al. [22] reported hernia recurrence rates of 23%, but an SSO up to 57% when a simultaneous panniculectomy was performed. Similarly, Elhage et al. [23] reported hernia recurrence rates of 8%, similar when panniculectomy is not performed. They also showed a higher SSO rate (45%). Performing a simultaneous panniculectomy in patients with an abdominal apron during ventral hernia repair may be feasible and carries an acceptable level of risk for SSOs and other complications. This approach not only provides excellent surgical visibility but is also, with the right training, well within the capabilities of a general surgeon. Opting for this combined procedure can potentially prevent the need for future surgeries and offers patients benefits such as improved self-esteem, mobility, and independence. Key to the success of AWR with panniculectomy is patient optimization, which involves a focus on preoperative weight loss, effective management of diabetes, smoking cessation, and enhanced respiratory function [32].

## 4. Discussion

For optimal surgery outcomes, it is crucial to prepare patients adequately, especially in emergency situations. The primary goal of AWR is to durably correct or alleviate hernias or abdominal wall defects, thereby reducing discomfort and enhancing daily activities for patients. Achieving a robust, innervated, and mesh-reinforced musculofascial coaptation is vital for dynamic and functional AWR.

Obese patients commonly exhibit abdominal wall hernias and defects and are at a higher surgical risk due to concurrent comorbidities. As mentioned previously, these patients have a higher rate of SSOs. Obesity contributes to a chronic, low-grade general inflammation due to a metabolic surplus, leading to excessive inflammatory responsiveness, oxidative stress, and immunosuppression, which impairs wound healing [30]. Obese patients struggle to cope with the surgical stress response from a metabolic standpoint. Additionally, the relatively poor perfusion of excessive subcutaneous fat leads to lower oxygen tension, further hindering wound healing. Diabetes or a hyperglycemic status, common in obese patients, also complicates wound healing [29]. Higher BMIs correlate with increased visceral fat, raising intra-abdominal pressure and complicating AWR, potentially leading to abdominal compartment syndrome. A previous study identified a BMI threshold of 31.9 kg/m^2^ above which the rate of SSO significantly increases [4].

For these reasons, many surgeons view obesity as a contraindication to performing AWR and, particularly, concomitant panniculectomy due to the elevated risk of wound healing complications and potential increase in hernia recurrence rates. Instead, the preoperative optimization of obese patients is crucial to mitigate surgical complications and hernia recurrence [4,32,33,34,35].

Modifiable risk factors should be addressed before performing AWR and/or panniculectomy. Tobacco use should be discontinued at least four weeks prior to surgery, though 12 weeks is recommended [36]. Each cigarette smoked reduces tissue oxygenation and perfusion, impairing wound healing and more than doubling the risk of infection [37]. Diabetes and hyperglycemia, often underappreciated conditions, need optimal management. A recent pooled analysis highlighted an increased risk of wound infection when preoperative glycated hemoglobin levels exceed 6–7% [36]. Surgeons should consider delaying elective AWR to improve glycemic control and reduce infection risk. Patient nutritional status is also important. Sarcopenia, a syndrome characterized by a generalized, progressive loss of muscle mass and strength, leading to functional impairment, is increasingly common in obese patients. Termed sarcopenic obesity, it is associated with high body fat, low skeletal muscle, and a high BMI [38]. Preoperative sarcopenia in AWR is linked to an increased risk of postoperative complications and a significantly higher rate of hernia recurrence [39]. To reduce morbidity and mortality, serum prealbumin levels should exceed 20 mg/dL, and serum albumin levels should be above 3.5 g/dL [40]. Reducing obesity itself, as it is linked to higher SSO and hernia recurrence, is also vital [4,22,23,24,25,26,27,28,29,30,31,32]. Medically supervised weight loss before surgery might reduce complication risk, and bariatric surgery prior to AWR can benefit morbidly obese patients [41]. Particularly, collaboration with a medical weight loss specialist using a modified protein sparing fast resulted in meaningful weight loss prior to complex abdominal wall reconstruction, and the majority of patients were able to maintain their weight loss during long-term follow-up [42].

In this context, panniculectomy can be a beneficial adjunct procedure for selected obese or previously obese patients, provided they have no significant comorbidities, to minimize complication risks.

While minimally invasive approaches for AWR are gaining popularity due to potentially lower SSO and shorter hospital stays, open AWR has undeniable advantages in cases requiring complex hernia dissections, secondary procedures, and larger defects. In these cases, redundant skin and subcutaneous tissue can be resected with panniculectomy (Figure 1), reducing subcutaneous dead space, enhancing vascularity of dissected skin flaps, and improving the aesthetic contour [6].

Finally, as obese patients are at increased risk of a venous thromboembolism [43], early postoperative mobilization is essential to prevent it, and effective pain control can facilitate ambulation. The intraoperative transversus abdominis plane block can reduce postoperative pain [44]. Minimizing narcotics and opioids is recommended; instead, local anesthetic pain catheters or liposomal bupivacaine can reduce opioid needs and shorten hospital stays [45,46].

Preoperative preparation, the incidence of obstructive sleep apnea (OSA), and the duration of surgery are crucial factors in the context of AWR procedures. However, it is noteworthy that the included studies in this review have not reported data on these aspects. Adequate preoperative preparation plays a vital role in optimizing surgical outcomes, particularly in complex procedures like AWR. This includes patient optimization through medical management of comorbidities, nutritional support, smoking cessation, and psychological assessment to ensure readiness for surgery. Lack of data on preoperative preparation in the included studies limits our understanding of its impact on surgical outcomes and potential variations in patient outcomes based on preoperative management strategies.

OSA is increasingly recognized as a significant risk factor for surgical complications, including respiratory compromise, cardiovascular events, and prolonged recovery times. In the context of AWR, where patients may already have compromised respiratory function due to obesity or other comorbidities, the presence of OSA can further exacerbate perioperative risks. Data on the incidence of OSA among patients undergoing AWR would provide valuable insights into risk stratification and perioperative management strategies tailored to this patient population.

The duration of surgery is an important determinant of perioperative outcomes, including the risk of surgical site infections, intraoperative complications, and overall patient recovery. Prolonged surgical duration may increase the risk of intraoperative complications, blood loss, and postoperative morbidity. Conversely, shorter surgical durations are generally associated with better outcomes and reduced healthcare costs. Understanding the typical duration of AWR procedures and its variability among different patient populations, particularly obese patients, and the addition of a panniculectomy can inform surgical planning, resource allocation, and perioperative risk assessment.

Some studies offer large sample sizes, long-term follow-up, and, notably, good comparability through a propensity score analysis [6,8,9,10,11,12,13,14,15,16,17,18,19,20,21,22,23,24,25,26,27,28,29,30,31,32]. Surgical techniques and mesh types are well described in some studies [6], though in many cases, a mesh was not used, indicating smaller defects. However, the impact of invasive procedures, such as component separation, has not been fully considered, which might significantly increase wound healing issues.

The current included studies are constrained by several limitations, notably the considerable heterogeneity observed within the patient population regarding baseline characteristics, comorbidities, types of hernias, and abdominal wall defects. Moreover, the variability in surgical approaches employed across different studies exacerbates this heterogeneity. Factors such as the type and size of mesh utilized, as well as variations in surgical techniques (such as component separation), study duration, and lengths of follow-up, introduce significant potential for bias in the reported outcomes. These multifaceted variables underscore the complexity of evaluating the effectiveness of treatments for hernias and emphasize the need for standardized methodologies to enhance comparability and reliability across studies. Further studies on this topic are warranted in order to improve the indications for these procedures.

## 5. Conclusions

Obesity complicates the process of AWR. Performing a panniculectomy concurrently with AWR is associated with a higher incidence of surgical site morbidity. However, it appears not to significantly impact the rate of hernia recurrence. While panniculectomy poses a higher risk of SSO in obese patients, it is crucial to thoroughly consider and communicate this approach with patients, given the heightened risk of wound healing complications.

## Figures and Tables

**Table 1:** PICOS criteria for inclusion and exclusion of studies.

Parameter	Inclusion Criteria	Exclusion Criteria
Patients	Patients of any age undergoing abdominal wall reconstruction and panniculectomy, particularly with obesity	Patients with other types of abdominal wall procedures
Intervention	Concomitant panniculectomy during abdominal wall reconstruction (AWR)	Studies not involving panniculectomy in AWR
Comparator	Any type of control, internal, external, or not	
Outcomes	Primary outcome measure: hernia recurrence at follow-up. Secondary outcome measures: any surgical site occurrence (SSO) at follow-up	
Study design	Randomized controlled trials; non-randomized observational trials; retrospective, prospective, or concurrent cohort studies	Reviews, expert opinion, comments, letter to editor, case reports, studies on animals, conference reports. Studies with no outcomes reported

## Data Availability

No new data were created or analyzed in this study. Data sharing is not applicable to this article.

## References

[1] Reilly J.J., El-Hamdouchi A., Diouf A., Monyeki A., Somda S.A. (2018). Determining the worldwide prevalence of obesity. Lancet.

[2] Chooi Y.C., Ding C., Magkos F. (2019). The epidemiology of obesity. Metabolism.

[3] Stenberg E., Cao Y., Szabo E., Näslund E., Näslund I., Ottosson J. (2018). Risk Prediction Model for Severe Postoperative Complication in Bariatric Surgery. Obes. Surg..

[4] Giordano S.A., Garvey P.B., Baumann D.P., Liu J., Butler C.E. (2017). The Impact of Body Mass Index on Abdominal Wall Reconstruction Outcomes: A Comparative Study. Plast. Reconstr. Surg..

[5] Poulose B.K., Shelton J., Phillips S., Moore D., Nealon W., Penson D., Beck W., Holzman M.D. (2012). Epidemiology and cost of ventral hernia repair: Making the case for hernia research. Hernia.

[6] Giordano S., Garvey P.B., Baumann D.P., Liu J., Butler C.E. (2017). Concomitant Panniculectomy Affects Wound Morbidity but Not Hernia Recurrence Rates in Abdominal Wall Reconstruction: A Propensity Score Analysis. Plast. Reconstr. Surg..

[7] Sosin M., Termanini K.M., Black C.K., Thanik V., Saadeh P.B., Levine J.P. (2020). Simultaneous Ventral Hernia Repair and Panniculectomy: A Systematic Review and Meta-Analysis of Outcomes. Plast. Reconstr. Surg..

[8] Moreno-Egea A., Campillo-Soto Á., Morales-Cuenca G. (2016). Does abdominoplasty add morbidity to incisional hernia repair? A randomized controlled trial. Surg. Innov..

[9] Mazzocchi M., Dessy L.A., Ranno R., Carlesimo B., Rubino C. (2011). “Component separation” technique and panniculectomy for repair of incisional hernia. Am. J. Surg..

[10] Zemlyak A.Y., Colavita P.D., El Djouzi S., Walters A.L., Hammond L., Hammond B., Tsirline V.B., Getz S., Heniford B.T. (2012). Comparative study of wound complications: Isolated panniculectomy versus panniculectomy combined with ventral hernia repair. J. Surg. Res..

[11] Berry M.F., Paisley S., Low D.W., Rosato E.F. (2007). Repair of large complex recurrent incisional hernias with retromuscular mesh and panniculectomy. Am. J. Surg..

[12] McNichols C.H., Diaconu S., Liang Y., Ikheloa E., Kumar S., Kumar S., Nam A., Rasko Y. (2018). Outcomes of ventral hernia repair with concomitant panniculectomy. Ann. Plast. Surg..

[13] Robertson J.D., de la Torre J.I., Gardner P.M., Grant J.H., Fix R.J., Vásconez L.O. (2003). Abdominoplasty repair for abdominal wall hernias. Ann. Plast. Surg..

[14] Saxe A., Schwartz S., Gallardo L., Yassa E., Alghanem A. (2008). Simultaneous panniculectomy and ventral hernia repair following weight reduction after gastric bypass surgery: Is it safe?. Obes. Surg..

[15] Cheesborough J.E., Dumanian G.A. (2015). Simultaneous prosthetic mesh abdominal wall reconstruction with abdominoplasty for ventral hernia and severe rectus diastasis repairs. Plast. Reconstr. Surg..

[16] Shermak M.A. (2006). Hernia repair and abdominoplasty in gastric bypass patients. Plast. Reconstr. Surg..

[17] Reid R.R., Dumanian G.A. (2005). Panniculectomy and the separation-of-parts hernia repair: A solution for the large infraumbilical hernia in the obese patient. Plast. Reconstr. Surg..

[18] Espinosa-de-los-Monteros A., Avendaño-Peza H., Gómez- Arcive Z., Martin-del-Campo L., Navarro-Navarro J. (2016). Total abdominal wall reconstruction with component separation, reinforcement, and vertical abdominoplasty in patients with complex ventral hernias. Aesthetic Plast. Surg..

[19] Downey S.E., Morales C., Kelso R.L., Anthone G. (2005). Review of technique for combined closed incisional hernia repair and panniculectomy status post-open bariatric surgery. Surg. Obes. Relat. Dis..

[20] Fischer J.P., Tuggle C.T., Wes A.M., Kovach S.J. (2014). Concurrent panniculectomy with open ventral hernia repair has added risk versus ventral hernia repair: An analysis of the ACS-NSQIP database. J. Plast. Reconstr. Aesthet. Surg..

[21] Hutchison C.E., Rhemtulla I.A., Mauch J.T., Broach R.B., Enriquez F.A., Hernandez J.A., Messa C.A., Williams N.N., Harbison S.P., Fischer J.P. (2019). Cutting through the fat: A retrospective analysis of clinical outcomes, cost, and quality of life with the addition of panniculectomy to ventral hernia repair in overweight patients. Hernia.

[22] Diaconu S.C., McNichols C.H.L., AlFadil S., Liang Y., Bai J., Silverman R.P., Grant M.P., Nam A.J., Rasko Y.M. (2019). Postoperative Outcomes in Obese Patients That Undergo Ventral Hernia Repair versus Ventral Hernia Repair with Concurrent Panniculectomy. Plast. Reconstr. Surg..

[23] Elhage S.A., Marturano M.N., Deerenberg E.B., Shao J.M., Prasad T., Colavita P.D., Kercher K.W., Heniford B.T., Augenstein V.A. (2021). Impact of panniculectomy in complex abdominal wall reconstruction: A propensity matched analysis in 624 patients. Surg. Endosc..

[24] Owei L., Swendiman R.A., Kelz R.R., Dempsey D.T., Dumon K.R. (2017). Impact of body mass index on open ventral hernia repair: A retrospective review. Surgery.

[25] Pernar L.I.M., Pernar C.H., Dieffenbach B.V., Brooks D.C., Smink D.S., Tavakkoli A. (2017). What is the BMI threshold for open ventral hernia repair?. Surg. Endosc..

[26] Regner J.L., Mrdutt M.M., Munoz-Maldonado Y. (2015). Tailoring surgical approach for elective ventral hernia repair based on obesity and National Surgical Quality Improvement Program outcomes. Am. J. Surg..

[27] Zolin S.J., Tastaldi L., Alkhatib H., Lampert E.J., Brown K., Fafaj A., Petro C.C., Prabhu A.S., Rosen M.J., Krpata D.M. (2020). Open retromuscular versus laparoscopic ventral hernia repair for medium-sized defects: Where is the value?. Hernia.

[28] Sharbaugh M.E., Patel P.B., Zaman J.A., Ata A., Feustel P., Singh K., Singh T.P. (2021). Robotic ventral hernia repair: A safe and durable approach. Hernia.

[29] Kudsi O.Y., Chang K., Bou-Ayash N., Gokcal F. (2021). A comparison of robotic mesh repair techniques for primary uncomplicated midline ventral hernias and analysis of risk factors associated with postoperative complications. Hernia.

[30] Maxwell D.O.D., Losken A., Elwood D. (2022). A Suture-less Underlay Ventral Herniorrhaphy Technique for High-Risk Complex Abdominal Wall Reconstruction. Am. Surg..

[31] Podolsky D., Ghanem O.M., Tunder K., Iqbal E., Novitsky Y.W. (2022). Current practices in complex abdominal wall reconstruction in the Americas: Need for national guidelines?. Surg. Endosc..

[32] Slater K., Ajjikuttira A.A. (2022). Is simultaneous panniculectomy an ideal approach to repair a ventral hernia: A general surgeon’s experience. Hernia.

[33] Rangel-Huerta O.D., Pastor-Villaescusa B., Gil A. (2019). Are we close to defining a metabolomic signature of human obesity? A systematic review of metabolomics studies. Metabolomics.

[34] Biancari F., Giordano S. (2019). Glycated Hemoglobin and the Risk of Sternal Wound Infection After Adult Cardiac Surgery: A Systematic Review and Meta-Analysis. Semin. Thorac. Cardiovasc. Surg..

[35] Khansa I., Janis J.E. (2019). The 4 Principles of Complex Abdominal Wall Reconstruction. Plast. Reconstr. Surg. Glob. Open.

[36] Jiménez-Ruiz A.C., Martín V., Alsina-Restoy X., Granda-Orive I.J., de Higes-Martínez E., García-Rueda M., Genovés-Crespo M., López-García C., Lorza-Blasco J.J., Márquez F.L. (2020). Cost-benefit analysis of funding smoking cessation before surgery. Br. J. Surg..

[37] Sørensen L.T. (2012). Wound healing and infection in surgery: The pathophysiological impact of smoking, smoking cessation, and nicotine replacement therapy: A systematic review. Ann. Surg..

[38] Stoklossa C.A.J., Sharma A.M., Forhan M., Siervo M., Padwal R.S., Prado C.M. (2017). Prevalence of Sarcopenic Obesity in Adults with Class II/III Obesity Using Different Diagnostic Criteria. J. Nutr. Metab..

[39] Barnes L.A., Li A.Y., Wan D.C., Momeni A. (2018). Determining the impact of sarcopenia on postoperative complications after ventral hernia repair. J. Plast. Reconstr. Aesthet. Surg..

[40] Loftus T.J., Brown M.P., Slish J.H., Rosenthal M.D. (2019). Serum Levels of Prealbumin and Albumin for Preoperative Risk Stratification. Nutr. Clin. Pract..

[41] Chandeze M.M., Moszkowicz D., Beauchet A., Vychnevskaia K., Peschaud F., Bouillot J.-L. (2019). Ventral hernia surgery in morbidly obese patients, immediate or after bariatric surgery preparation: Results of a case-matched study. Surg. Obes. Relat. Dis..

[42] Rosen M.J., Aydogdu K., Grafmiller K., Petro C.C., Faiman G.H., Prabhu A. (2015). A Multidisciplinary Approach to Medical Weight Loss Prior to Complex Abdominal Wall Reconstruction: Is it Feasible?. J. Gastrointest. Surg..

[43] Venclauskas L., Maleckas A., Arcelus J.I., ESA VTE Guidelines Task Force (2018). European guidelines on perioperative venous thromboembolism prophylaxis: Surgery in the obese patient. Eur. J. Anaesthesiol..

[44] Oppenheimer A.J., Fiala T.G.S., Oppenheimer D.C. (2016). Direct Transversus Abdominis Plane Blocks With Exparel During Abdominoplasty. Ann. Plast. Surg..

[45] Morales R., Mentz H., Newall G., Patronella C., Masters O. (2013). Use of abdominal field block injections with liposomal bupivicaine to control postoperative pain after abdominoplasty. Aesthet. Surg. J..

[46] Giordano S., Uusalo P., Oranges C.M., di Summa P.G., Lankinen P. (2020). Local anesthetic pain catheters to reduce opioid use in massive weight loss patients undergoing abdominoplasty: A comparative study. J. Plast. Reconstr. Aesthet. Surg..

